# Real-World Treatment Patterns and Survival in Uveal Melanoma: A Multicenter Cohort Study by the Turkish Oncology Group (TOG)

**DOI:** 10.3390/cancers18030394

**Published:** 2026-01-27

**Authors:** Sercan On, Selin Cebeci, Zeynep Hande Turna, Zeynep Gülsüm Güç Sevgen, Deniz Can Guven, Sadettin Kılıçkap, Mehmet Nuri Başer, Bilgin Demir, Sedat Biter, Ertuğrul Bayram, Berkay Yeşilyurt, Doğan Uncu, Ahmet Melih Arslan, Elif Atağ Akyürek, Hayati Arvas, Zuhat Urakçı, Teoman Şakalar, Ferit Aslan, Mehmet Uzun, Mustafa Yıldırım, Ahmet Unlu, Derya Kıvrak Salim, Atike Pınar Erdoğan, Elif Sahin, Şeyda Gündüz, Burcu Gülbağcı, Şaziye Burçak Karaca Yayla, Burcu Cakar

**Affiliations:** 1 Department of Medical Oncology, Ege University Faculty of Medicine, İzmir 35100, Türkiye; sercan.on@ege.edu.tr (S.O.);; 2 Department of Medical Oncology, Cerrahpaşa Faculty of Medicine, İstanbul University–Cerrahpaşa, İstanbul 34098, Türkiye; 3 Department of Medical Oncology, İzmir Katip Çelebi University Atatürk Training and Research Hospital, İzmir 35620, Türkiye; 4 Department of Medical Oncology, Hacettepe University, Ankara 06230, Türkiye; 5 Department of Medical Oncology, İstinye University Faculty of Medicine, İstanbul 34010, Türkiye; 6 Department of Medical Oncology, Aydın Adnan Menderes University Faculty of Medicine, Aydın 09010, Türkiye; 7 Department of Medical Oncology, Çukurova University Faculty of Medicine, Adana 01250, Türkiye; 8 Department of Medical Oncology, Ankara Bilkent City Hospital, Ankara 06520, Türkiye; 9 Department of Medical Oncology, Dokuz Eylul University Faculty of Medicine, İzmir 35340, Türkiye; 10 Dokuz Eylul University Institute of Oncology, İzmir 35340, Türkiye; 11 Department of Medical Oncology, Dicle University Faculty of Medicine, Diyarbakır 21280, Türkiye; 12 Department of Medical Oncology, Kahramanmaraş Sütçü İmam University Faculty of Medicine, Kahramanmaraş 46000, Türkiye; 13 Medical Park Ankara Hospital, Ankara 06510, Türkiye; 14 Department of Medical Oncology, Tepecik Training and Research Hospital, İzmir 35100, Türkiye; 15 Department of Medical Oncology, Sanko University Faculty of Medicine, Gaziantep 27310, Türkiye; 16 Department of Medical Oncology, University of Health Sciences Antalya Training and Research Hospital, Antalya 07070, Türkiye; 17 Department of Medical Oncology, Manisa Celal Bayar University Faculty of Medicine, Manisa 45100, Türkiye; 18 Department of Medical Oncology, Kocaeli City Hospital, Kocaeli 41200, Türkiye; 19 Department of Medical Oncology, Koç University Hospital, İstanbul 34300, Türkiye; 20 Department of Medical Oncology, Tekirdağ City Hospital, Tekirdağ 59000, Türkiye

**Keywords:** melanoma, uveal, ciliary body melanoma, nivolumab, ipilimumab, tebentafusp, local therapies

## Abstract

**Background/Objectives**: Uveal melanoma (UM) is a subtype of melanoma that originates from the uveal layer of the eye (iris, ciliary body, choroid) and is biologically distinct from cutaneous melanoma. The real-world evidence on treatment patterns and survival outcomes, particularly from non-Western populations, remains limited. This study provides a comprehensive national overview of local and systemic management of UM across Türkiye. **Methods**: This multicenter retrospective cohort study, coordinated by the Turkish Oncology Group (TOG), included patients diagnosed with UM between 2012 and 2025 across 19 tertiary oncology centers. Clinical characteristics, treatment patterns, and outcomes were analyzed. Systemic therapies were subgrouped as immunotherapy or cytotoxic chemotherapy. Progression-free survival (PFS) and overall survival (OS) were assessed by the Kaplan–Meier method; prognostic factors for OS were analyzed by Cox regression. **Results**: A total of 113 patients were included; 89.4% presented with localized disease. During follow-up, 43.4% developed metastases, predominantly hepatic (93.5%). 47.4% of metastatic patients received liver-directed treatments; the most common treatment was radioembolization, and these treatments were associated with longer OS. Among 59 patients receiving systemic therapy, immunotherapy demonstrated higher ORR and DCR than chemotherapy across treatment lines, although small subgroup sizes limited statistical significance. Dual immune checkpoint blockade emerged as an independent favorable prognostic factor in multivariate analysis. Median OS for metastatic patients was 16 months (95% CI: 9.3–22.7). HLA-A*02:01 was positive in 29.4% of patients tested, and only three patients received tebentafusp. **Conclusions**: This large national cohort highlights substantial real-world heterogeneity in treatment patterns and confirms the limited efficacy of chemotherapy, the prognostic benefit of dual ICI therapy, and the potential survival advantage of liver-directed treatments in selected patients. Low HLA-A*02:01 positivity and limited access to tebentafusp remain major challenges in Türkiye. These findings provide an essential benchmark for improving treatment strategies in metastatic UM.

## Simple Summary

Uveal melanoma is a rare but aggressive malignancy with a strong tendency to metastasize, particularly to the liver. Once metastasis occurs, treatment options are limited, and survival outcomes remain poor. This multicenter study evaluates real-world outcomes of patients with metastatic uveal melanoma treated across various institutions in Türkiye. We examined both systemic treatment options, including immune checkpoint inhibitors, chemotherapy, and targeted therapies, as well as local treatment approaches, such as liver-directed therapies. By analyzing their effectiveness and survival impact in routine clinical practice, our findings provide a comprehensive overview of current therapeutic strategies. This study offers valuable insights to help clinicians optimize treatment selection and highlights areas where further research and improved therapeutic approaches are needed.

## 1. Introduction

Uveal melanoma (UM) is the most common primary intraocular malignancy in adults, accounting for 3–5% of all melanomas and affecting 5–7 individuals per million annually [1]. UM originates from any component of the uveal tract, but the vast majority arise in the choroid or ciliary body, whereas iris tumors account for only a small minority [2]. Most patients present with localized disease; fundoscopic examination can detect the lesion. Although it is not mandatory to perform a biopsy to confirm the diagnosis, a biopsy is recommended to enable molecular testing for the patient. Staging was performed according to the AJCC 8th edition criteria for uveal melanoma [3].

Primary UM is typically managed with surgery or radiotherapy, both of which achieve excellent local control in contemporary practice [1]. Although local recurrence is uncommon, metastatic spread remains frequent, typically emerging within a few years of initial treatment, with occasional relapses occurring more than a decade later [4]. The liver is the most frequent, and often the sole, site of metastasis, and unlike cutaneous melanoma, lymph node and brain involvement is uncommon [5]. This makes liver-directed locoregional therapies a rational strategy; however, while improving hepatic disease control, these approaches have not consistently translated into a meaningful survival advantage [6].

Metastatic UM carries a poor prognosis, with a median survival typically around one year and fewer than 15% of patients alive at five years [7]. Tebentafusp, a gp100-targeted TCR–CD3 bispecific agent, is the only systemic therapy proven to extend overall survival in a randomized trial, increasing median survival to nearly two years, but its benefit remains limited to HLA-A*02:01–positive patients [8]. For HLA-A*02:01–negative patients, therapy relies mainly on immune checkpoint inhibitors (ICIs), although responses remain modest and evidence derives primarily from non-randomized studies [9,10,11,12]. Importantly, much of the existing evidence in metastatic UM is based on small observational cohorts or phase II studies, and robust randomized data remain limited. Current literature is further constrained by heterogeneous treatment practices and the lack of comprehensive real-world datasets that capture the whole clinical trajectory from diagnosis through multiple lines of therapy, including the interplay between local and systemic treatments.

To address these knowledge gaps, this multicenter study, coordinated by the Turkish Oncology Group (TOG) and conducted across 19 tertiary oncology centers, presents a nationwide real-world evaluation of UM. By incorporating data from both localized and metastatic disease settings, the study provides a comprehensive assessment of treatment patterns, surveillance outcomes, and the effectiveness of systemic therapies in routine clinical practice.

## 2. Materials and Methods

This multicenter retrospective cohort study, coordinated by the Turkish Oncology Group (TOG), included patients with histopathologically or clinically confirmed uveal melanoma who were evaluated, followed, or treated at 19 tertiary oncology centers between January 2012 and June 2025. Patients receiving only local treatment or surveillance were also included. Follow-up and surveillance of patients managed in the localized disease setting were conducted according to the routine clinical practice and institutional protocols of each participating center. Patients who were never referred to medical oncology clinics, were managed entirely outside oncology centers, or were diagnosed with an active concurrent malignancy were excluded. June 2025 served as the data cutoff. A minimum contribution of two patients per center was required for inclusion in the study. Patient contribution varied across participating centers, ranging from two to 13 patients per center.

Baseline demographic and clinical characteristics were summarized using descriptive statistics. Categorical variables were reported as frequencies and percentages, while continuous variables were presented as means with standard deviation (SD) or medians with interquartile range (IQR), as appropriate. Group comparisons were performed using the chi-square or Fisher’s exact test for categorical variables and Student’s *t*-test or Mann–Whitney U test for continuous variables.

Treatment response was assessed using RECIST v1.1. Objective response rate (ORR) was defined as the proportion of patients who achieved a complete or partial response, and disease control rate (DCR) was defined as the proportion who achieved a complete response, a partial response, or stable disease.

Systemic treatments were grouped into immunotherapy (nivolumab, ipilimumab, nivolumab–ipilimumab, pembrolizumab, tebentafusp) and cytotoxic chemotherapy. Treatment efficacy was evaluated across all applicable therapy lines.

Overall survival (OS) was defined as the time from metastatic diagnosis to death or last follow-up, and progression-free survival (PFS) as the time from systemic therapy initiation to progression or death. Survival outcomes were estimated using the Kaplan–Meier method and compared using the log-rank test. Prognostic factors for OS were analyzed using Cox proportional hazards models, with results expressed as hazard ratios (HRs) and 95% confidence intervals (CIs). Variables included in the multivariate Cox regression model were predefined a priori based on established clinical relevance in the literature and data availability across centers [3,6,7,10].

All statistical analyses were conducted using SPSS version 27 (IBM Corp., Armonk, NY, USA). A two-sided *p*-value < 0.05 was considered statistically significant. The study was approved by the institutional review boards of all participating centers and conducted in accordance with the Declaration of Helsinki. Informed consent was waived due to the retrospective study design.

During the editing of the manuscript, the authors used ChatGPT 5.2 (OpenAI) exclusively for language editing, grammar correction, and improving clarity of expression. The tool was not used for data analysis, study design, result interpretation, generation of scientific content, or creation of figures/tables. The authors entirely produced all scientific interpretations, analyses, and conclusions. The authors reviewed and verified all AI-assisted text and take full responsibility for the final content.

## 3. Results

### 3.1. All Cohort

A total of 113 patients were included in the study. Of these, 62 patients (54.9%) were male, and the mean age was 56.2 years (SD ±12.2). At diagnosis, 101 patients (89.4%) presented with localized disease, whereas 12 (10.6%) had de novo metastatic disease. Baseline characteristics are summarized in Table 1. The choroid or ciliary body was the most common primary tumor site (71.7%), followed by the iris (7.1%), while tumor localization was not reported in 21.2% of cases. Most melanomas arose de novo, whereas six patients (5.3%) developed melanoma from a pre-existing nevus.

With respect to local treatment, enucleation was performed in 67 patients (59.3%), plaque brachytherapy in 40 (35.4%), and six patients (5.3%) did not receive any local treatment. Radiotherapy was additionally administered to 27 patients, primarily for positive surgical margins or local recurrence. Among the 101 patients under surveillance following local treatment, the median follow-up was 42.8 months (IQR, 22.1–79.8). Over the course of follow-up, four patients (4%) experienced isolated local recurrence, all treated with salvage radiotherapy, whereas 49 patients (48.5%) developed distant metastases.

**Table 1:** All patient characteristics.

	All Patients N (%)
Age (years)	56.2 (SD ± 12.2)
Sex	
Male	62 (54.9)
Female	51 (45.1)
T category	
Tx	38 (33.6)
T1	13 (11.5)
T2	26 (23)
T3	19 (16.8)
T4	17 (15.1)
N category	
Nx	20 (17.7)
N0	88 (77.9)
N1	4 (3.5)
M category	
M0	101 (89.4)
M1	12 (10.6)
Tumor localization	
Choroid and ciliary body	81 (71.7)
Iris	8 (7.1)
Unknown	24 (21.2)
Primary treatment	
Enucleation	67 (59.3)
Plaque brachytherapy	40 (35.4)
No local treatment	6 (5.3)
Recurrence	
No	48 (47.5)
Local	4 (4)
Distant	49 (48.5)

Abbreviations: SD, standard deviation; Tx, primary tumor cannot be assessed; T1–T4, tumor size categories; Nx, regional lymph nodes cannot be assessed; N0, no regional lymph node metastasis; N1, regional lymph node metastasis; M0, no distant metastasis; M1, distant metastasis.

### 3.2. Metastatic Cohort

Sixty-one patients had metastatic disease, including 12 (10.7%) with de novo metastases and 49 (43.4%) who developed metastases during follow-up. The liver was the predominant site of metastasis (93.5%), with isolated extrahepatic disease observed in only four patients (6.5%). Among patients with liver involvement, 70% had ≥4 lesions and 75% demonstrated bilobar distribution. The most common extrahepatic metastatic sites were the lung (20.4%), bone (18.4%), and lymph nodes (18.4%).

Patients with de novo and recurrent metastatic disease did not differ significantly in terms of age, sex, or baseline disease characteristics (Table 2). Among the 57 patients with liver metastases, 27 (47.4%) underwent liver-directed treatment, most commonly transarterial radioembolization (44.4%), followed by radiofrequency ablation (29.6%), surgical resection or hepatic arterial infusion (each 11.1%), and radiotherapy (3.2%). In most cases, locoregional therapy was administered as first-line treatment (25 of 27 patients). The number of procedures varied, with 14 patients treated once, seven twice, and the remainder three or more times, reaching up to ten procedures in a single patient. Approximately one-third of patients received concurrent systemic therapy, most commonly chemotherapy. The median PFS following liver-directed treatment was 4.1 months (95% CI, 2.8–5.2), and the addition of concurrent systemic therapy did not result in a significant improvement in PFS (log-rank *p* = 0.43).

HLA-A*02:01 testing was performed in 17 patients, of whom 5 (29.4%) were positive.

**Table 2:** Characteristics of metastatic patients.

	All Metastatic Patients	Recurrence Metastatic Patient	Denovo Metastatic Patient N (%)	*p* Value
Age (years)				
Median (IQR)	56 (47–64)	54 (44.5–63.5)	61.5 (53.7–67.5)	*p* = 0.072
Sex				
Male	37 (60.7)	29 (59.2)	8 (66.7)	*p* = 0.75
Female	24 (39.3)	20 (40.8)	4 (33.3)	
Metastatic site				
Isolated hepatic	37 (60.7)	31 (63.3)	6 (50)	*p* = 0.70
Isolated extrahepatic	4 (6.5)	3 (6.1)	1 (8.3)	
Both	20 (32.8)	15 (30.6)	5 (41.7)	
Hepatic metastasis number				
1–3 lesions	17 (29.8)	16 (34.8)	1 (9.1)	*p* = 0.08
≥4 lesions	40 (70.2)	30 (65.2)	10 (90.9)	
Hepatic involvement pattern				
Unilobar	14 (24.6)	12 (26.1)	2 (20)	*p* = 0.52
Bilobar	43 (75.4)	34 (73.9)	8 (80)	
HLA 02*01 status				
Positive	5 (8.2)	5 (10.2)	0 (0)	N/A
Negative	12 (19.7)	11 (22.4)	1(8.3)	
Unknown	44 (72.1)	33 (67.3)	11 (91.7)	

Abbreviations: IQR, interquartile range; HLA, human leukocyte antigen.

### 3.3. First-Line Treatment

**Figure 1:**
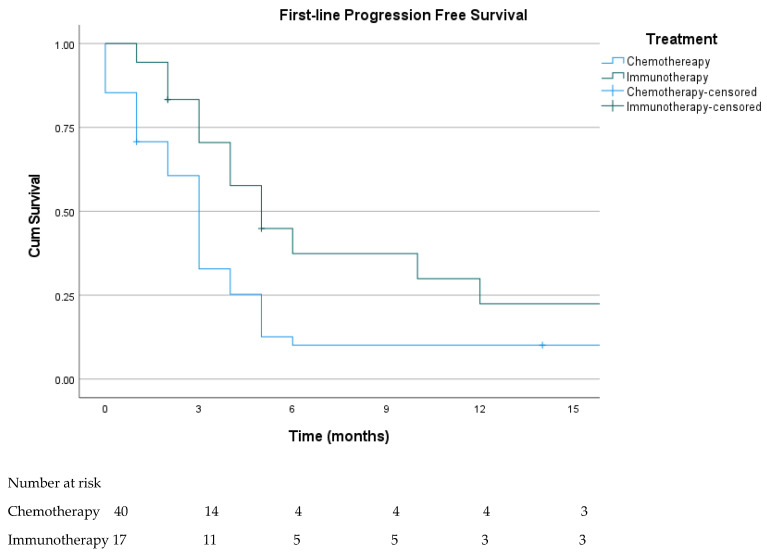
Kaplan–Meier analysis of PFS according to first-line treatment (chemotherapy vs. immunotherapy). Median PFS was 5.3 months (95% CI, 3.1–6.9) for the immunotherapy group and 3.1 months (95% CI, 2.4–3.5) for the chemotherapy group (log-rank *p* = 0.007). Numbers at risk at 3-month intervals up to 15 months are shown below the curves.

Two patients with metastatic disease died before initiation of systemic therapy, whereas 59 received at least one line of treatment. The median follow-up among treated patients was 11.5 months (IQR: 2.7–24.7). In the first-line setting, most patients received cytotoxic chemotherapy (41/59, 69.5%), predominantly temozolomide (49.2%) or carboplatin/paclitaxel (18.6%). Immunotherapy was administered to 18 patients (30.5%), including nivolumab (n = 10), ipilimumab (n = 2), nivolumab–ipilimumab (n = 5), and tebentafusp (n = 1). Immunotherapy was administered to 18 patients (30.5%), including nivolumab (n = 10), ipilimumab (n = 2), nivolumab–ipilimumab (n = 5), and tebentafusp (n = 1). Although ORR (38.9%) and DCR (55.6%) were numerically higher in the immunotherapy group than in the chemotherapy group, the differences did not reach statistical significance (Table 3). Median PFS was significantly longer with immunotherapy (5.3 months) compared with chemotherapy (3.1 months), as shown in Figure 1 (log-rank *p* = 0.007). The single patient treated with tebentafusp achieved a partial response and remained progression-free at six months.

### 3.4. Second-Line Treatment

**Figure 2:**
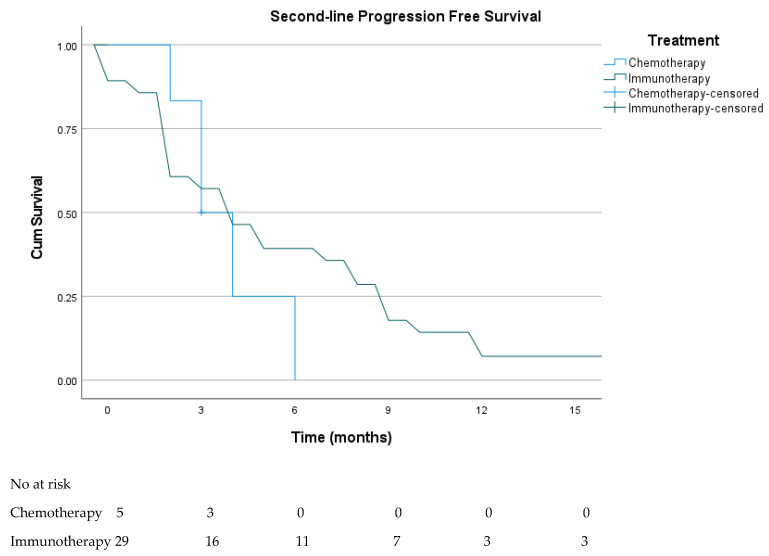
Kaplan–Meier PFS curves according to second-line treatment (chemotherapy vs. immunotherapy). The median PFS was 4.4 months (95% CI: 1.9–6.9) in the immunotherapy group and 3.2 months (95% CI: 1.6–4.4) in the chemotherapy group (log-rank *p* = 0.40). Numbers at risk at 3-month intervals up to 15 months are shown below the curves.

A total of 36 patients received second-line therapy, of whom 30 (83.3%) were treated with immunotherapy (nivolumab n = 24, ipilimumab n = 2, nivolumab–ipilimumab n = 4), while only six patients (16.7%) received chemotherapy. Among those treated with immunotherapy, the ORR and DCR were 26.7% and 46.7%, respectively, whereas no objective responses were observed in the chemotherapy cohort (DCR 16.7%). These differences were not statistically significant (*p* = 0.30 and *p* = 0.36). Median PFS was 4.4 months (95% CI, 1.9–6.9) for immunotherapy and 3.2 months (95% CI, 1.6–4.4) for chemotherapy, with no statistically significant difference observed between groups (log-rank *p* = 0.40; Figure 2).

### 3.5. Later-Line Treatment

Later-line therapy was administered to 19 patients, including chemotherapy in 11 (57.9%), immune checkpoint inhibitors in four (21%), tebentafusp in two (10.5%), and olaparib in one patient with a RAD54 mutation. Across all third-line treatments, the ORR was 15.8% and the DCR was 52.6%, with a median PFS of 4.1 months (95% CI, 2.8–5.2).

Among the two patients treated with tebentafusp, one achieved stable disease and remained on treatment for five months, while the other achieved a partial response but progressed after 13 months. Seven patients received fourth-line therapy, with no further treatments administered thereafter. A partial response was observed in one patient treated with ipilimumab, while no other objective responses were recorded.

### 3.6. Overall Survival

**Figure 3:**
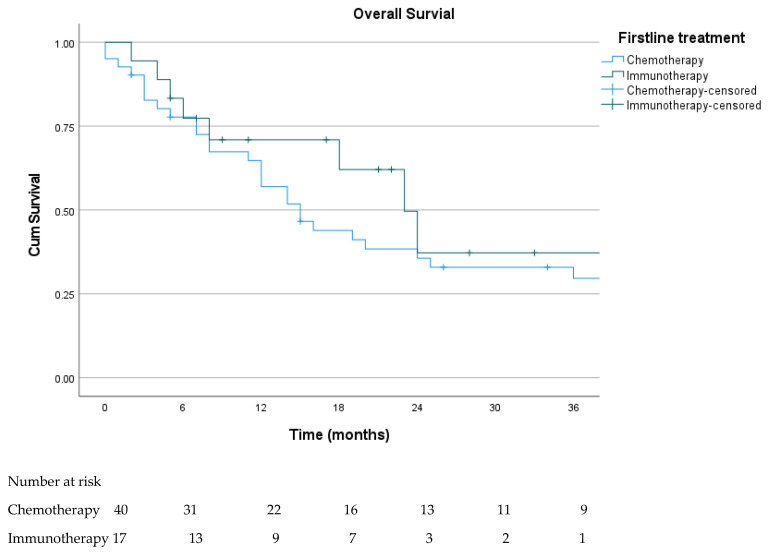
Kaplan–Meier OS curves according to first-line treatment (chemotherapy vs. immunotherapy). The median OS was 23.0 months (95% CI, 15.9–30.1), versus 15.0 months (95% CI, 10.2–19.8). Numbers at risk at 6-month intervals up to 36 months are shown below the curves.

All metastatic patients were included in the Kaplan–Meier analysis. At the time of data cutoff, 18 patients (29.5%) were alive. Median overall survival (OS) was 16.0 months (95% CI: 9.3–22.7). In patients who were able to access immunotherapy in the first-line setting, median overall survival was 23.0 months (95% CI, 15.9–30.1), versus 15.0 months (95% CI, 10.2–19.8) in patients treated with first-line chemotherapy, with no statistically significant difference observed (log-rank *p* = 0.544) (Figure 3).

In univariate analysis, the presence of multiple liver metastases and extrahepatic involvement was associated with significantly worse OS (*p* = 0.02 for each), whereas liver-directed therapy and combination immunotherapy administered at any treatment line were associated with improved OS (*p* < 0.001 and *p* = 0.03, respectively). In multivariate analysis, local liver-directed therapy (HR 0.40, 95% CI, 0.19–0.84; *p* = 0.02) and combination immunotherapy (HR 0.31, 95% CI, 0.10–0.95; *p* = 0.04) remained independent favorable prognostic factors (Table 4).

## 4. Discussion

In this large multicenter national cohort, consistent with global epidemiologic trends, most patients presented with localized disease [1]. The choroid and ciliary body were the predominant primary sites (71.7%), while iris tumors were uncommon (7.1%), mirroring international patterns [2]. A notable proportion of missing T and N classifications limited complete AJCC staging, an essential constraint given the wide prognostic variation across stages [13]. Although plaque brachytherapy is the preferred local modality worldwide, enucleation remained the most frequently used approach in our cohort, likely reflecting institutional practice differences and treatment availability [14].

Despite effective local therapy, almost half of the patients eventually developed distant metastases, reflecting the well-known long-term relapse risk in uveal melanoma [5]. Monosomy 3, BAP1 mutations, and gene expression profiling class are recognized indicators of aggressive disease and increased recurrence risk in localized uveal melanoma, but these data were unavailable in our cohort [15]. Hepatic involvement was observed in 93.5% of patients with recurrent disease, consistent with the well-recognized hepatic tropism of UM [5]. A high liver metastatic burden (≥4 lesions) was present in nearly 70% of patients and was associated with poorer overall survival on univariate analysis, but this association was not retained after multivariate adjustment. Additional metastatic sites included the lung, bone, and lymph nodes, which are consistent with the literature [5].

Multiple liver-directed approaches have demonstrated improvements in response rates and PFS in phase II–III studies (HAI [16], TACE [17], radioembolization [18,19], IHP [6], PHP [20]), though a consistent overall survival benefit remains uncertain [7,21]. In our study, nearly half of the metastatic patients received liver-directed therapy, most commonly radioembolization, and this group demonstrated significantly longer overall survival, suggesting a potential benefit in real-world practice. However, as treatment allocation was not randomized, patients selected for liver-directed approaches were likely those with more favorable clinical characteristics, including better performance status and lower extrahepatic disease burden; therefore, these findings should be interpreted cautiously and should not be considered evidence of a causal treatment effect. Given the heterogeneity of liver-directed modalities and varying institutional practices, the observed survival benefit likely reflects outcomes in selected patients rather than a uniform treatment effect and should not be generalized across all liver-directed treatments. Evidence regarding the addition of concurrent systemic therapy remains limited. A recent CHOPIN trial reported improved PFS with nivolumab–ipilimumab combined with percutaneous hepatic perfusion; in contrast, our small subgroup treated concurrently did not show a clear advantage [22].

For many years, metastatic UM was managed with conventional chemotherapy, yet response rates consistently remained below 10%, and no survival benefit was demonstrated [23,24]. Our findings similarly confirmed the limited activity of cytotoxic agents. Uveal melanoma differs fundamentally from cutaneous melanoma, being primarily driven by *GNA11* and *GNAQ* mutations that activate MAP kinase and YAP1 signaling pathways [25] and characterized by a low tumor mutational burden and an immunologically “cold” microenvironment [26]. These features likely underlie the limited efficacy of ICIs reported in the literature, with single-agent ICI achieving objective response rates below 10% and a median overall survival of approximately 10 months [10]. In our cohort, immunotherapy was predominantly administered as a single agent in the second and subsequent treatment lines, mainly due to reimbursement constraints. Although immunotherapy resulted in numerically higher ORR and DCR than chemotherapy, these differences were not statistically significant, which may partly be due to the limited sample size. Kaplan–Meier analysis did not demonstrate a statistically significant difference in OS between patients receiving first-line immunotherapy and those treated with chemotherapy, indicating that treatment sequence alone may not be an important determinant of survival in this population. However, the immunotherapy-treated subgroup was heterogeneous, including different immunotherapy strategies with distinct efficacy profiles, which represents a substantial limitation for interpreting our findings.

By simultaneously targeting complementary immune inhibitory pathways, dual blockage with anti-PD-1 and anti-CTLA-4 may partially overcome intrinsic immune resistance. Dual ICI has shown modest but superior activity compared with monotherapy in previous phase II trials, with median PFS of 3.0–5.5 months and OS of 12.7–19.1 months [9,27]. More recent evidence reinforces this pattern: a large systematic review of 1414 patients reported limited efficacy of ICIs overall but consistently better outcomes with dual therapy [10], and a real-world comparison showed higher ORR and longer PFS with nivolumab–ipilimumab versus anti–PD-1 alone, despite similarly limited OS (12–13 months) [11]. While doublet ICI was associated with improved survival in multivariate analysis in our cohort, the limited number of treated patients and the likelihood of preferential treatment of clinically fitter individuals warrant cautious interpretation.

Tebentafusp, a bispecific T-cell engager that redirects polyclonal T cells to gp100-expressing melanoma cells via CD3 binding and induces tumor cell apoptosis independently of tumor mutational burden, represents a significant therapeutic advance in metastatic uveal melanoma [8]. However, its global use remains limited by access constraints and ethnic variability in HLA-A*02:01 prevalence. Reported frequencies range from ~40–50% in European/North American populations to ~20% in South American cohorts and as low as 10–15% in African ancestry groups, highlighting substantial geographical disparities in eligibility [28]. Although Türkiye-specific data are lacking, our study provides valuable early insight: among the 17 patients tested, only 5 (29.4%) were HLA-A*02:01-positive. Consequently, only three patients received tebentafusp, preventing meaningful assessment of treatment outcomes. Real-world evidence suggests a potential benefit when sequencing tebentafusp with immune checkpoint inhibitors, and phase II data in previously treated patients report a median OS of 16.8 months [29,30]. Additionally, a propensity-score–adjusted analysis demonstrated superior OS with tebentafusp compared with nivolumab–ipilimumab in the first-line setting, reinforcing its role as the preferred frontline option where available [31].

In meta-analyses prior to the standard use of tebentafusp, the median survival was around 12 months, while in studies after tebentafusp, a median survival of approximately 20 months was achieved [32,33]. In our study, with the limited access of new treatments to patients, a median survival of 16 months was obtained, which is better than traditional survival but lower than studies where all patients had access to tebentafusp.

This study, due to its retrospective design, includes selection bias related to patient registration methods, institutional practices, and reimbursement arrangements that may differ across centers. Metastatic surveillance and follow-up strategies are limited to patients referred only to medical oncology clinics and, therefore, may not fully represent the population-based sample. Additionally, detailed information regarding treatment doses, modifications, durations, and toxicity in these patients could not be clearly obtained from the registry data. Because of the limited number of patients receiving doublet ICI, the small number of events, and missing performance status data, the multivariate analyses are underpowered and should be regarded as exploratory. Despite these limitations, this study provides one of the most comprehensive real-world datasets on uveal melanoma in our region.

## 5. Conclusions

In this large multicenter real-world cohort, nearly half of patients with localized uveal melanoma eventually developed metastatic disease, with the liver remaining the dominant site. Liver-directed therapy and doublet ICI were independently associated with improved overall survival, highlighting their role in selected patients. Chemotherapy demonstrated limited efficacy, whereas immunotherapy provided modest but clinically meaningful benefit. The low rate of HLA-A*02:01 positivity and restricted access to tebentafusp underscore important challenges for implementing the only therapy with a proven survival advantage. Overall, these findings help clarify current treatment patterns in Türkiye and emphasize the need for broader access to effective systemic therapies and prospective studies to optimize management strategies for metastatic uveal melanoma.

## Figures and Tables

**Table 3:** Objective Responses and Disease Control by Treatment Line and Modality.

Treatment Line	Treatment Type	CR n (%)	PR n (%)	SD n (%)	ORR %	DCR %	*p*-Value
First-line (N = 59)	Chemotherapy (n = 41)	1 (2.4)	7 (17.1)	4 (9.8)	19.5	29.3	*p* = 0.19/0.05
	Immunotherapy (n = 18)	0 (0)	7 (38.9)	3 (16.7)	38.9	55.6	
Second-line (N = 36)	Chemotherapy (n = 6)	0 (0)	0 (0)	1 (16.7)	0	16.7	*p* = 0.30/0.36
	Immunotherapy (n = 30)	1 (3.3)	7 (23.3)	6 (20.0)	26.7	46.7	

Abbreviations: CR, complete response; PR, partial response; SD, stable disease; ORR, objective response rate; DCR, disease control rate. ORR is calculated as (CR + PR)/total number of patients. DCR is calculated as (CR + PR + SD)/total number of patients. For each treatment line, the first *p*-value refers to the comparison of ORR between chemotherapy and immunotherapy, while the second *p*-value refers to the comparison of DCR between the two groups.

**Table 4:** Univariate and Multivariate Analysis Results (OS).

Variable	Univariate Analysis HR (95% CI)	*p* Value	Multivariate Analysis HR (95% CI)	*p* Value
Age (per year)	1.01 (0.98–1.04)	*p* = 0.28	1.01 (0.98–1.04)	*p* = 0.56
Male sex	1.45 (0.77–2.74)	*p* = 0.25	1.56 (0.81–3.01)	*p* = 0.18
Multiple liver metastases (≥4 lesions)	2.33 (1.13–4.23)	***p*** ** = 0.02**	2.01 (0.85–4.75)	*p* = 0.11
Extra-hepatic metastases	2.20 (1.16–4.17)	***p*** ** = 0.02**	1.20 (0.56–2.58)	*p* = 0.64
Local liver-directed therapy	0.35 (0.18–0.66)	***p*** ** < 0.001**	0.40 (0.19–0.84)	***p*** ** = 0.02**
Combination immunotherapy	0.37 (0.15–0.96)	***p*** ** = 0.03**	0.31 (0.10–0.95)	***p*** ** = 0.04**

**Bold values** indicate statistically significant results.

## Data Availability

The data supporting the findings of this study are available from the corresponding author upon reasonable request. The dataset is not publicly available due to institutional and ethical restrictions.

## Abbreviations

The following abbreviations are used in this manuscript:

AJCC	American Joint Committee on Cancer
CI	Confidence interval
CR	Complete response
DCR	Disease control rate
HR	Hazard ratio
ICI	Immune checkpoint inhibitor
IHP	Isolated hepatic perfusion
IQR	Interquartile range
IRB	Institutional Review Board
N/A	Not applicable
ORR	Objective response rate
OS	Overall survival
PFS	Progression-free survival
PHP	Percutaneous hepatic perfusion
PR	Partial response
RECIST	Response Evaluation Criteria in Solid Tumors
SD	Stable disease
SPSS	Statistical Package for the Social Sciences
TACE	Transarterial chemoembolization
TARE	Transarterial radioembolization
TOG	Turkish Oncology Group
UM	Uveal melanoma
